# Alpha and beta band correlates of haptic perceptual grouping: Results from an orientation detection task

**DOI:** 10.1371/journal.pone.0201194

**Published:** 2018-07-19

**Authors:** Antonio Prieto, Julia Mayas, Soledad Ballesteros

**Affiliations:** Studies on Aging and Neurodegenerative Diseases Research Group, Departamento de Psicología Básica II, Facultad de Psicología, Universidad Nacional de Educación a Distancia, Madrid, España; Universität Regensburg, GERMANY

## Abstract

Behavioral and neurophysiological findings in vision suggest that perceptual grouping is not a unitary process and that different grouping principles have different processing requirements and neural correlates. The present study aims to examine whether the same occurs in the haptic modality using two grouping principles widely studied in vision, spatial proximity and texture similarity. We analyzed behavioral responses (accuracy and response times) and conducted an independent component analysis of brain oscillations in alpha and beta bands for haptic stimuli grouped by spatial proximity and texture similarity, using a speeded orientation detection task performed on a novel haptic device (*MonHap*). Behavioral results showed faster response times for patterns grouped by spatial proximity relative to texture similarity. Independent component clustering analysis revealed the activation of a bilateral network of sensorimotor and parietal areas while performing the task. We conclude that, as occurs in visual perception, grouping the elements of the haptic scene by means of their spatial proximity is faster than forming the same objects by means of texture similarity. In addition, haptic grouping seems to involve the activation of a network of widely distributed bilateral sensorimotor and parietal areas as reflected by the consistent event-related desynchronization found in alpha and beta bands.

## Introduction

Perceptual grouping refers to “the fact that observers perceive some elements of the visual field as going together more strongly than others” [1]. Research efforts addressing the specific principles that determine how perceptual grouping occurs and the mechanisms that govern its operation have focused primarily on vision and audition, where grouping principles have been relatively well established [1,2]. To date, however, little research has been conducted to investigate whether these perceptual grouping principles also apply to the sense of touch. There are, at least, three main reasons: First, the early claims about the lack of applicability of the Gestalt principles to touch [3]; second, the serial nature of tactile exploration, and third, difficulties related to the controlled presentation of the stimuli and response registration. Even reviews of tactile perception have scarcely touched on the topic [4,5]. However, in the last decade, there has been renewed interest in this topic [6], guided both by the theoretical interest in knowing the shared and/or specific mechanisms behind grouping in different sensory modalities, and the potential practical applications that a better knowledge of how the tactile perceptual scene is organized into meaningful objects may have in several areas. Among these areas are the design of visuo-tactile/haptic displays and interfaces, and the development of tactile resources and substitution devices for visually impaired people.

Specifically, Chang et al. [7] used visual and haptic layouts composed of 7 to 16 squares that vary in proximity and similarity (color/texture) and asked their participants to indicate the number of groups and the reason they based their response. The researchers found that participants grouped visual and tactile patterns in a similar way. The same occurred when participants had to group the elements using the Gestalt principle of continuation [8]. More recently, Overvliet et al. [9,10] conducted two studies in which participants either explored haptic random dot displays (contour detection) or columns composed of vertical and horizontal lines (haptic search). The authors demonstrated that proximity and similarity grouping principles influence haptic contour detection and haptic search respectively. Finally, Verlaers et al. [11] and Overvliet and Plaisier [12], using haptic enumeration tasks in which participants were asked to count tangible dots while moving their finger pads over a tactile display, showed that both proximity and configural grouping cues can speed up haptic enumeration. These convergent results suggest the existence of similar underlying mechanisms in visual and haptic perceptual grouping. However, these similarities should be taken with caution, given the fundamental differences in how the sensory information is acquired in each sensory modality. Particularly, in vision the information processing is largely parallel. In contrast, the acquisition of sensory information during haptic exploration is sequential (which involves active movements not totally under experimental control) and may involve other cognitive processes that are not present in visual tasks (e.g., the activation of working memory processes to keep haptic information available during the exploration).

A fundamental question is whether perceptual grouping is a unitary process that underlies all grouping principles [13]. Traditional views assume that grouping is an early single process along the cognitive stream, consisting of combining similar types of tokens into larger ones, and to construct the boundaries between different sets, a process that provides the units which other perceptual processes will use as input [14,15]. Nonetheless, the empirical evidence challenges the notion that grouping is a simple and unitary phenomenon. On this line, findings indicate that perceptual grouping is formed by, at least two different processes: 1) a unit formation and clustering process responsible for determining which elements belong together and the segregation from other elements, and 2) a shape formation, or configuring process, involved in the global appearance of the grouped elements depending on their interrelations. This processes would take place at different processing stages, and might have different cognitive requirements [13,16]. This view implies not only that different grouping principles implicate different cognitive resources and neurological correlates [17], but also that the time needed to group the elements of the perceptual scene will be different depending on the features (spatial arrangement, shape, texture) in which the grouping process is based.

The latter question has been specifically addressed in the visual modality by comparing proximity and similarity grouping principles. Results suggest that behavioral responses to stimuli grouped by proximity are faster than to those grouped by similarity, and that electrophysiological substrates and processing requirements would be different in the two principles [18]. More specifically, Han and colleagues [19,20] found that the perception of stimuli was faster and more accurate when they were grouped by spatial proximity than by color/shape similarity, and that both principles followed distinct neural pathways (dorsal/ventral stream, respectively). Moreover, according to Mao et al. [21], the faster behavioral responses to stimuli grouped by proximity in vision are due to the fact that proximity grouping modulates activity in the primary visual cortex early (60–90 ms), whereas similarity grouping does not. These results suggest that the formation of objects based on the spatial relationships of their constituent elements is faster than that based on the characteristics that are common to those elements.

Only a few visual studies have addressed the neural basis of perceptual grouping from the time frequency perspective, and their methodologies and results are mixed. For example, Volberg et al. [22] found increased beta-band power over occipito-parietal sites in a contour detection task when the participants were able to perceive the contour. Also, Aissani et al. [23] reported increased beta-band activity in centro-parietal sites when stimuli where perceived as a whole in a form/motion perceptual integration task. By contrast, Zaretskaya and Bartels [24] found decreased beta-band power over posterior parietal sites associated with global Gestalt perception and perceptual grouping. This contrasting pattern of results could be explained by the distinction between local and global processing. For example, Romei et al. [25] found that bursts of right-parietal Transcranial magnetic stimulation (TMS) at beta and theta frequency benefited local and global processing respectively. Thus, the increased beta power in the first two studies could indicate the existence of more local processing requirements.

Grouping studies in the haptic modality have focused mainly on the behavioral study of the applicability of grouping principles to touch and their influence over other cognitive processes [7–12,26]. However, there is no direct empirical evidence that accounts for both the behavioral and the neural correlates of perceptual grouping in touch [6], but see Blankenburg [27] for an indirect approximation to the study of the neural correlates of haptic grouping.

In the present study, we investigated the behavioral (RTs and accuracy) and electrophysiological correlates (time/frequency oscillatory brain activity) of spatial proximity and texture similarity for stimuli presented to touch without vision. We focused on transient event-related spectral perturbations (ERSP) of alpha and beta bands over sensorimotor and parietal regions, a measure of the event-related shifts in the power spectrum during the task period. The power changes within these frequency bands constitute the predominant activity of the so-called μ sensorimotor rhythms (SMR) [28]. Furthermore, given that previous studies have shown the involvement of visual areas in haptic processing [29,30], we also focused on alpha band activity within the occipital cortex as an indicator of the activity of visual areas when exploring stimuli by touch. To avoid the confound derived from the mixed EEG signals recorded from the scalp (which include contributions from different brain sources), we employed independent component analysis (ICA) and clustering methods [31] instead of the raw data from scalp electrodes to decompose the EEG recorded signal into the maximally temporally independent signals available in the data channels (also called independent components -ICs). Then, we performed the brain activity analyses on the resultant ICs. This separation and identification of independent brain sources is essential to characterize the neuropsychological origins of the brain processes, and to relate a specific task with the activity and topography of those brain sources [32]. We used a touch-adapted speeded orientation detection task similar to the one used in vision by Han [18]. To present the haptic stimuli and record the participants’ responses, we used a specifically designed haptic device (MonHap) adapted from an apparatus originally designed to investigate lateralization in haptic processing in monkeys [33]. This apparatus enabled us to control the presentation of the haptic stimuli and to record EEG activity.

The aims of the current study were twofold: (1) To examine the behavioral differences in speed and accuracy of these two grouping principles in the haptic modality; and (2) to investigate the brain oscillatory activity of grouping by proximity and grouping by similarity for stimuli presented by touch.

Given that previous studies [7–11] have shown that proximity and similarity grouping principles seem to operate similarly in vision and touch, we hypothesized that orientation would be detected faster and more accurately in patterns grouped by proximity compared to those grouped by similarity.

Regarding IC-cluster analysis of brain oscillations (ERSP), we expected the recruitment of a large bilateral sensorimotor network, reflected in the power reduction (event-related desynchronization or ERD) of alpha and beta bands over contra- and ipsi-lateral sensorimotor and parietal cortices. In addition, we hypothesized that activity within this network would increase in the grouping by similarity condition relative to proximity grouping, especially in sensory integration areas, due to the need to compute two different textures and to integrate this information into a unified percept. As mentioned above, previous studies have shown the contribution of visual areas in haptic processing, especially in object recognition [29,30,34], so our aim was also to investigate the involvement of occipital visual areas in haptic perceptual grouping.

## Methods

### Participants

Fifteen (11 females) volunteer students at the Universidad Nacional de Educación a Distancia (UNED) participated in the study. Their mean age was 34 years (SD = 10.06; range 20–49). All participants reported being right-handed, had normal tactile perception and were naïve to the purpose of the experiment. They signed an informed consent form for participation in the study, which was approved by the Ethical Committee of the UNED. The experiment was conducted in accordance with the ethical standards laid down in the 1964 Declaration of Helsinki as revised in October 2008.

### Apparatus and stimuli

The *MonHap* haptic device was used for stimulus presentation and data collection. The device consists of an electromagnetically shielded (to avoid possible artifacts) opaque box with two apertures to introduce the hands and two platforms containing an array of 10 x 10 small holes in which the cylinders were plugged to create the desired configuration. The haptic device was interfaced with two computers. The first controlled the stimulus presentation and recorded exploration times and accuracy. The second recorded the EEG data.

**Fig 1:**
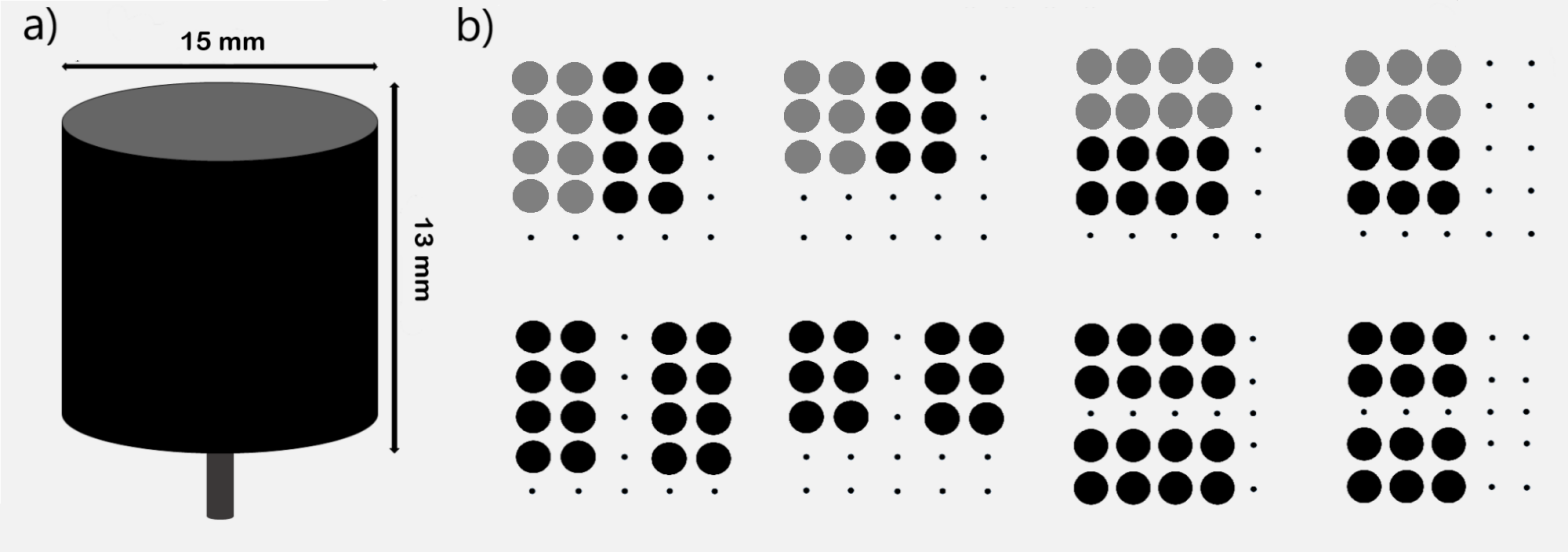
Individual stimuli and patterns used in the orientation detection task. (A) An overview of the individual cylinders used to form the different haptic patterns. (B) Stimulus configurations used in the orientation detection task. The top and bottom rows of Fig 1B show the patterns used for similarity and proximity conditions respectively. The left and right side of Fig 1B shows vertical and horizontal stimuli respectively. In similarity condition (top row), the grey and black circles represent the rough and smooth textures employed for texture-similarity grouping.

The stimuli consisted of 24 touch-sensitive cylinders measuring approximately 13mm (height) x 15mm (diameter) each, specifically designed for the experiment. Sixteen cylinders had a smooth metallic texture and the remaining had a rough texture created by covering the cylinder surface with sandpaper (see Fig 1A). The stimuli were arranged in patterns of 12 (4 x 3) or 16 (4 x 4) cylinders to form 12 different Gestalt grouped patterns oriented vertically/horizontally, that were matched in number in both grouping conditions (see Fig 1B). All haptic patterns were confined within a 5 x 5 (95 mm x 95 mm total exploration area) square matrix (a sub-matrix of the 10 x 10 array of the *MonHap* device). This arrangement ensured that the haptic exploration area was the same in all the experimental conditions. In the texture-similarity condition (Fig 1B upper row), the grey circles represent the rough textured cylinders, while the black circles represent the smooth textured cylinders. Thus, in the similarity condition, the two orientations were defined by the different textures that compound the haptic pattern (half rough and the other half smooth), while the distance between the different elements remain constant. On the contrary, in the proximity condition (Fig 1B bottom row), all the cylinders had the same texture (smooth in 50% of the proximity trials and rough in the other 50% of the trials), so participants cannot rely on texture differences to identify the orientation of the global pattern, and the orientation of the pattern was defined by the different spatial distance between the elements. Horizontal stimuli were those that, according to the grouping principle used (proximity or texture similarity), formed two rows of elements perpendicular to the mid-sagittal plane of the participant´s body (see Fig 1B, right). Vertical stimuli were those that formed two rows of elements parallel to the mid-sagittal plane of the participant´s body (see Fig 1B, left). In the similarity conditions, the gap between each single element was fixed at 6 mm (see Fig 1B, upper row), whereas in the proximity condition the distance between each element was 6 and 25 mm for close and far elements respectively (see Fig 1B, bottom row).

The experimental room was shielded to avoid electromagnetic artifacts from the outside. Lighting and temperature conditions were kept constant for all participants.

### Procedure

Participants were seated in an armchair facing the front of the MonHap at approximately 30 cm, with the midline of the body aligned with the center of the apparatus. After placing the EEG cap and preparing the electrodes, the experimenter indicated the participants to introduce their right (dominant) hand through the aperture located on the right side of the apparatus (and also located to the right relative to the body mid-line) and to become familiar with the presentation platform where the stimuli would be presented. To avoid muscular artifacts produced by head, body and arm movements, participants were encouraged to face forward during the experiment, to avoid head movements and maintain eye fixation and to support their backs on the seat and rest their forearm and the edge of their hand on the side of the presentation platform. They were also asked not to touch the stimuli before the start of a trial. Once the participants where comfortably seated, the experimenter gave detailed instructions to the participants about the task. The task consisted of detecting the orientation of patterns formed by means of spatial proximity (where orientation was determined by sets of elements that were spatially close relative to the others) and texture similarity (where orientation was determined by sets of elements with the same texture) grouping principles. Trials with “vertical” orientation were defined as those whose orientation was parallel to the mid-sagittal plane of the body. Trials with “horizontal” orientation were defined as those whose orientation were perpendicular to the mid-sagittal plane of the body. The patterns themselves, were presented in line with the transverse plane of the body (horizontal plane relative to the floor). The experimenter instructed the participants to decide on each trial whether the patterns were oriented vertically or horizontally relative to their body midline, according to the definitions given above, and execute the response as fast as possible but trying to avoid errors. Each trial began with the computer program randomly generating the next configuration and displaying it on a computer screen only visible for the experimenter. The experimenter then arranged the pattern generated, by plugging each individual cylinder in the correct position into the MonHap. Once the stimulus setup was completed, the experimenter guided the hand of the participant to a predesignated start position within the presentation platform, to ensure that all participants began the haptic exploration in the same conditions. Participants waited in the start position with the edge of their hand placed on the right side of the pattern (without touching it), until a green led light placed in front of the participant signaled the start of the trial. The participants, then, placed the hand over the haptic pattern and used their index, middle, ring and pinkie fingers to explore the pattern. Once they reached a decision about the orientation of the pattern, participants responded by pressing one of the two foot pedals (one for each orientation) that were counterbalanced across participants. This method ensured that all participants started the haptic exploration in the same way and explored the pattern using the same fingers. Moreover, although a green light indicated when to place the hand over the pattern and start the exploration, the actual beginning of each trial (in terms of response time measurement and event markers for EEG acquisition) was determined by an automatic signal sent to the computer at the first contact of the participant´s hand with the stimulus (cylinders were touch sensitive and sent the signal to the EEG computer immediately after the first contact with any cylinder). This allowed us to accurately control the extent of the haptic exploration and the response times for all participants, regardless of the delay between the onset of the led light and the first contact with the stimulus. Once the trial finished, the participants were instructed to rest the hand on the side of the presentation platform while the experimenter configured the next trial.

After a practice phase that ended only after participants perfectly understood the task and all the procedures, participants performed a total of 112 experimental trials divided into two blocks of 56 trials each with a 5-minute resting interval between blocks. Upon completion of the task, participants were debriefed.

### EEG pre-processing and epoch rejection

A 34-channel elasticized Quick-cap with Ag/AgCl sintered electrodes (Neuroscan Medical supplies, Inc.) was used to record EEG data from scalp electrodes (FP1, FP2, F7, F3, FZ, F4, F8, FT9, FT7, FC3, FCZ, FC4, FT8, FT10, T3, C3, CZ, C4, T4, TP7, CP3, CPZ, CP4, TP8, T5, P3, PZ, P4, T6, PO1, PO2, O1, OZ, O2) positioned according to the extended international 10–20 system (American EEG society, 1991). To control the influence of ocular artifacts, vertical (VEOG) and horizontal (HEOG) electro-oculograms were recorded in two bipolar channels. Eye blinks and vertical eye movements were monitored via electrodes located below and on the supra-orbital ridge of the left eye. Horizontal artifacts were monitored via electrodes on the outer canthus of each eye. Linked mastoids (A1 A2) were used as reference and participants were grounded to the AFz electrode. All data were digitized using a NuAmps amplifier (Neuroscan Inc.) in continuous recording mode. Sampling rate was 250Hz and all channels were online band-pass filtered (0.1-70Hz) and notch filtered (50Hz) to eliminate power line artifacts. The overall impedance was maintained below 5kΩ. Prior to the task, participants were shown their ongoing EEG on the computer screen to teach them how to avoid eye blinking, jaw clenching and body movement artifacts.

Offline data preprocessing and analysis of the EEG recordings was performed using the EEGLAB toolbox [35] and ERPLAB plugin for EEGLAB [36], both running under MATLAB environment (The MathWorks, Inc.). Continuous data were filtered offline using a digital FIR (finite impulse response) filter (1–30 Hz; 12 dB/oct. roll-off). Combination of low-high cutoffs and filter order (12 dB/oct. roll-off) were selected to increase statistical power by removing the maximum amount of noise while causing minimal distortion of the data (using the lowest order that eliminates low and high frequency artifacts without resulting in excessive attenuation of target frequencies). After filtering, data were separated into baseline corrected and non-overlapping epochs time-locked to the haptic exploration onset, ranging from 1000 ms before to 3000 ms after the first contact of the participant´s hand with the tactile pattern, with the pre-stimulus interval (1000 ms) as baseline period. Epoch rejection was performed in a semi-automated way. First, we visually inspected the epoched data to eliminate epochs containing high amplitude/frequency and other irregular artifacts, but retaining stereotypic artifacts in the data. We then conducted infomax extended ICA decomposition (see Section 3.2 for details) using the runica algorithm implemented in EEGLAB [37]. The activities and scalp maps of the resulting independent components (ICs) were then plotted and visually inspected to identify artifactual components. Low frequency and high amplitude components with scalp topographies centered over the VEOG and HEOG sites were noted and rejected as blink and eye-movement related artifacts respectively. Components with focal topographies and increased activity in the range of 20–30 Hz were noted and rejected as muscle activity (EMG) artifacts [38,39]. Then, we again visually inspected the pruned datasets and rejected any artifactual epochs remaining. Finally, a second ICA decomposition was performed on the artifact free epochs and the resulting weights were saved for averaging and posterior analyses. This resulted in an average of 96 ICA-pruned epochs per participant (min = 73, max = 104).

## Data analysis

### Analysis of behavioral data

We used two dependent measures to evaluate behavioral performance: (1) The mean response times (RTs) corresponding to correct responses computed as the time between the first contact with the stimuli and the participant’s response, and (2) accuracy of the orientation detection task.

Preliminary exploratory analysis of accuracy data showed deviations from a normal distribution in this dependent variable. Accuracy showed a pronounced positive skewness. We therefore transformed accuracy data (proportion of errors) by replacing proportions that were equal to 0 with *1/4n* (where *n* is the number of observations on which the proportion is estimated for each group) and then applying an Arcsine (angular) transformation *θ = sin*^*-1*^ *(√p)*, to fit normally distributed data on proportions and percentages that follow the binomial distribution [40].

Participants’ RTs above and below 3 times the standard deviation were removed from the analysis. Overall, 2.25 trials (range 0 to 4) were removed from each participant (2.14% of the total of valid trials).

### Independent component analysis (ICA) and component clustering

This study used EEGLAB to decompose the N channel EEG signal into N temporally independent components arising from distinct brain and non-brain sources [31,41]. According to the authors, the use of ICA to decompose the signal is based on two assumptions: (1) The EEG signal at a given electrode is a linear sum of temporally independent sources from spatially fixed locations; (2) Volume conduction does not involve significant time delays in the spatial spread of the electric current [32]. This linear combination can be reverted to find an unmixing matrix, **W**, in the equation **u** = **W**x, where **u** is the source matrix and **x** is the scalp EEG. We used the default extended-mode runica training parameters [37], an extension of the original algorithm of Bell and Sejnowski [31] and stopping weight change set to 1e – 7. The extended mode makes it possible to separate a wider range of source signals (both super- and sub-Gaussian) maintaining simplicity, while the conservative stopping learning criterion lengthens ICA training, enabling cleaner and more reliable decompositions, particularly with more than 33 channels and a limited number of epochs. After submitting epochs to ICA decomposition, artefactual components were removed by inspection of their scalp topography and spectral power as detailed in Section 2.4.

Spatial localization of the remaining ICs was analyzed using the DIPFIT2 toolbox [42] (available from sccn.ucsd.edu/eeglab/dipfit.html). This tool attempts to spatially locate the cortical source of a given IC by hypothesizing a dipole source that could generate the scalp map potential distribution, compute a forward model that accounts for the maximum amount of variance in the scalp map, and represent it by three-dimensional coordinates (x, y, z). For each IC, a best-fitting single equivalent dipole was localized using a boundary element head model (BEM) where electrode coordinates where warped into. Next, to identify similar ICs in the orientation detection task across participants, the ICs of the 12 subjects were grouped into clusters based on similar dipole locations, scalp topographies and event-related spectral perturbations (ERSPs) using the *STUDY* function of EEGLAB. In this study, the parameters of the clustering function were selected combining three different criteria: 1) The objectives and EEG measures selected; 2) the empirical evaluations of different parameter combinations along with recommendations from the EEGLAB developers and advanced users (which include using all types of information available, controlling the relative influence of each factor and keep the number of dimensions around 20); and 3) the clustering procedures employed in previous haptic studies using IC-clustering [43]. Prior to clustering, ICs outside the brain volume and those with residual variance above 30% were removed. For the remaining clusters, the clustering procedure was performed using the following steps: 1) To construct a common measure to specify the ‘distances’ (in a N-dimensional space) between ICs for their use by the clustering algorithm, scalp topography and ERSP were computed along with dipole source location; 2) ERSP and scalp topography measures were compressed and combined into a 10-dimensional vector each, due to the limitations of the EEGLAB pre-clustering algorithms and the redundancy of the data (e.g. around 3000 time/frequency ERSP values) using principal component analysis (PCA), while dipole location was combined into a 3-dimensional vector (x, y, z), resulting in a 23-dimensional combined position vector (that account for the ‘distances’ between ICs); 3) these measures were normalized by dividing the measure data of all PCAs by the standard deviation of the first principal component of the specific measure. The dipole location measure was then weighted by a factor of 10, ERSP measure by a factor of 3 and scalp topography by a factor of 5. Independent components more than 3 standard deviations away from cluster centroid were removed. Finally, the EEGLAB k-means algorithm was applied to the combined measure to obtain 23 maximally distinct clusters. As this study aimed to explore the brain processes implicated in haptic sensorimotor and perceptual processes, we identified and selected clusters located in left and right sensorimotor cortices that were characterized by the presence of μ rhythm (with spectral power peaks around 10 and 20 Hz), parietal located clusters characterized by α rhythm (8–14 Hz), as well as occipital α clusters (8–14 Hz), to examine the implication of motor, sensory, spatial and visual processes in haptic grouping. These clusters were selected according to their location (visual inspection of the equivalent dipoles and scalp maps, along with the Talairach coordinates of the cluster centroid) and the ERSP activity found during the task.

### Time frequency analysis and event-related spectral perturbations

To test the dynamics of the IC-clusters power spectra, epochs in each experimental condition were subjected to a time-frequency analysis in order to compute the event-related changes in power spectrum (ERSP) for each IC cluster over time [44]. All time-frequency analyses were performed on EEGLAB together with custom MATLAB scripts. Time windows were referenced to time 0, which denoted the first contact of the participant’s hand with the haptic stimulus. The time window started at -1000 ms and ended at 2996 ms. Baseline spectral power was computed in the [–1000–300] window. We removed the [–300 0] data interval to avoid including the perceptual effect derived by the onset of the green led that signals the start of the trial. After defining the window of interest, we computed the spectral power for each frequency, obtained the average across trials and plotted the results as relative changes in spectral log amplitudes from the baseline [45]. The ERSP was computed over 70 log-spaced frequencies (padding 8) from 8 Hz to 25 Hz and 200 time points. We used the complex Morlet wavelet approach [46], a method that provides a time-varying estimate of signal magnitude in each frequency band, offering a good compromise between time and frequency resolution, improving frequency resolution at higher frequencies compared to standard wavelet convolution [35]. The wavelet family used in this study consisted of 8 cycles at the lowest frequency (8 Hz) with an expansion factor of 0.5, progressively increasing the number of cycles to 12.5 cycles at the highest frequency (25 Hz). Finally, we used single-trial baseline correction to compute mean ERSP for each individual trial [47]. This method calculates for every trial and frequency, 200 complex vectors evenly spaced in time. The raw spectral power is calculated by squaring the length of this vectors. Then each power value is divided by the average power across trials in the baseline to obtain 200 power ratios. This power ratios are averaged to yield the mean proportional changes in spectral power relative to baseline. This proportion is finally log-transformed to obtain a measure of event related spectral changes in Decibels (dB) [48].

### Statistical analysis

Behavioral data were analyzed using repeated measures *t*-test to explore statistical differences between proximity and similarity grouping conditions in both RTs and error rates (error rates were arcsine transformed prior to the analyses to meet parametric test assumptions as detailed in Section 3.1).

To deal with the multidimensionality of EEG data and the multiple comparison problem (familywise error rate), statistical analyses on the ERSP of the selected IC-clusters were run using the Fieldtrip plug-in for MATLAB [49], with non-parametric cluster-based permutation/Monte Carlo statistics, to determine the significant differences between grouping conditions. Time/frequency characteristics of all component epochs were first split into 2 samples (A and B) corresponding to the two experimental conditions; then the difference in means between these two samples was calculated (the observed value **T**). Next, **A** and **B** particular values were divided into two groups of size ***n***_***A***_ and ***n***_***B***_ in every possible way (every permutation of the two groups) and difference in sample means was calculated for each permutation to obtain the distribution of possible differences under the null hypothesis that the group label does not matter. Finally, *p*-values were calculated as the proportion of sampled permutations where the difference in means was greater than or equal to **T.** Finally, given that the present study focused on the differences between grouping conditions within pre-defined cluster locations (and not on the relative importance of these clusters for haptic grouping), all the statistical analyses were performed between grouping conditions within each cluster. Therefore, statistical tests between clusters were not conducted.

## Results

### Behavioral results

#### Response time (RT)

**Fig 2:**
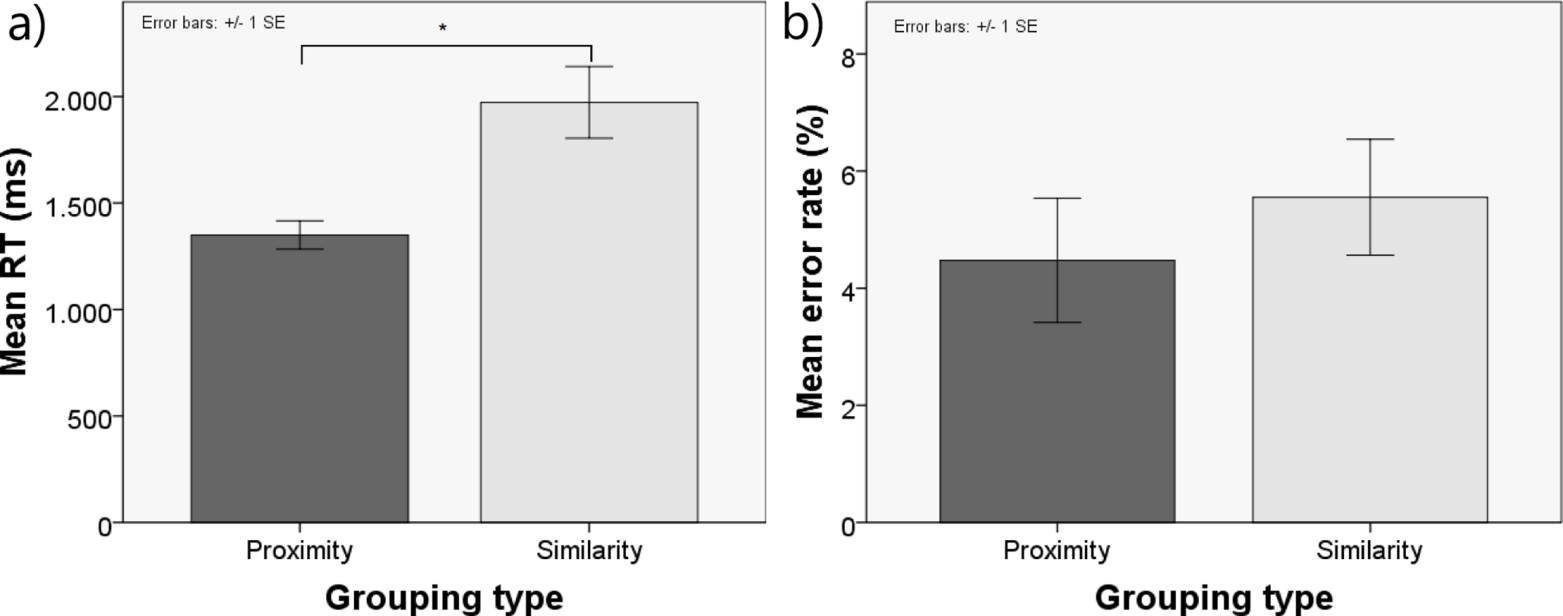
Behavioral results of the orientation detection task. Mean response times (A) and percentage of errors (B) in the orientation detection task as a function of grouping type. Error bars represent the standard error of the mean (SE) over participants.

A paired-samples t-test was conducted on RTs for correct responses. The t-test revealed statistically significant differences between proximity and similarity grouping conditions [*t* (1, 14) = -3.78; *p* = 0.002; *ƞ2p* = 0.506]. Participants detected the orientation of patterns faster when they were grouped by proximity (M = 1378 ms, SD = 222) than when they were grouped by texture similarity (M = 1920 ms, SD = 534) (see Fig 2A).

#### Accuracy

A paired-samples t-test was also performed on the arcsine-transformed proportion of errors. No significant differences were found between proximity (M = 0.044, SD = 0.342) and similarity (M = 0.059, SD = 0.395) grouping conditions in error rate (see Fig 2B).

### EEG dynamics

**Fig 3:**
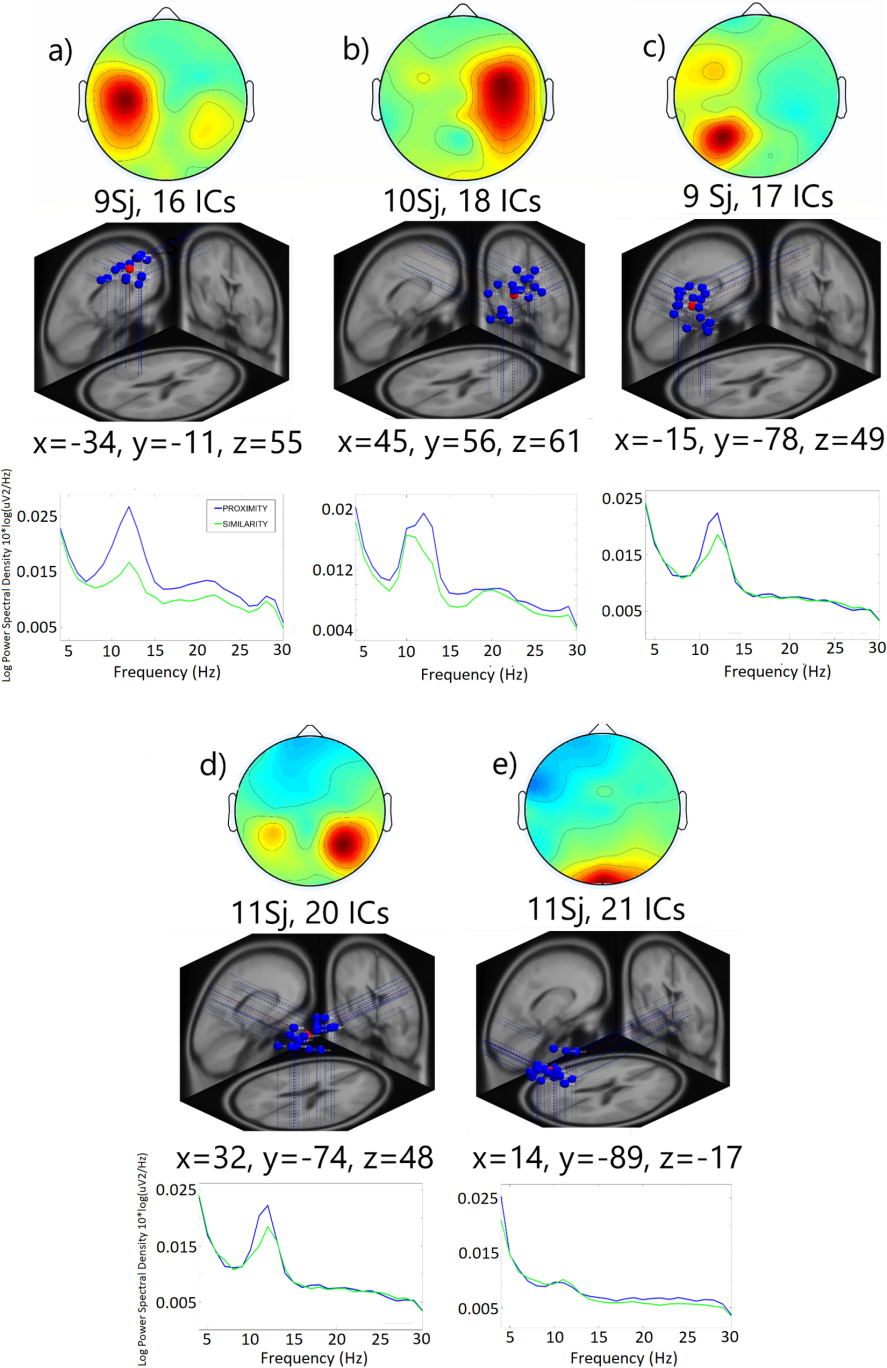
Characteristics of the 5 IC-clusters of interest. Scalp maps, number of participants and constituent ICs, dipole locations, cluster centroid coordinates [50], and power spectra for the 5 IC clusters of interest. From top-left to bottom right: a) left sensoriomotor, b) right sensoriomotor, c) left parietal, d) right parietal, e) occipital.

Three participants were eliminated from the EEG analyses due to the large number of artifacts, so all the EEG analyses were performed on the remaining 12 participants. We first removed bad channels and artefactual characteristics of the data, decomposed the remaining epoched EEG into spatially fixed and temporally independent components, eliminated artifactual ICs (eye and muscular artifacts) and fitted dipole models to the scalp topography of those components. The remaining ICs from the 12 participants (306 out of 432) were grouped into 23 clusters according to their dipole locations, scalp topographies and ERSP characteristics. This study aimed to explore the brain correlates of perceptual grouping in active touch, so in the following paragraphs, we will focus on and further analyze 5 component clusters located in or near left sensorimotor, right sensorimotor, left parietal, right parietal and occipital areas that showed relevant event-related spectral modulations during the task period. Fig 3 shows the scalp maps, dipole locations and power spectra of the selected components.

#### IC-cluster event-related spectral perturbation (ERSP) and power spectra

**Fig 4:**
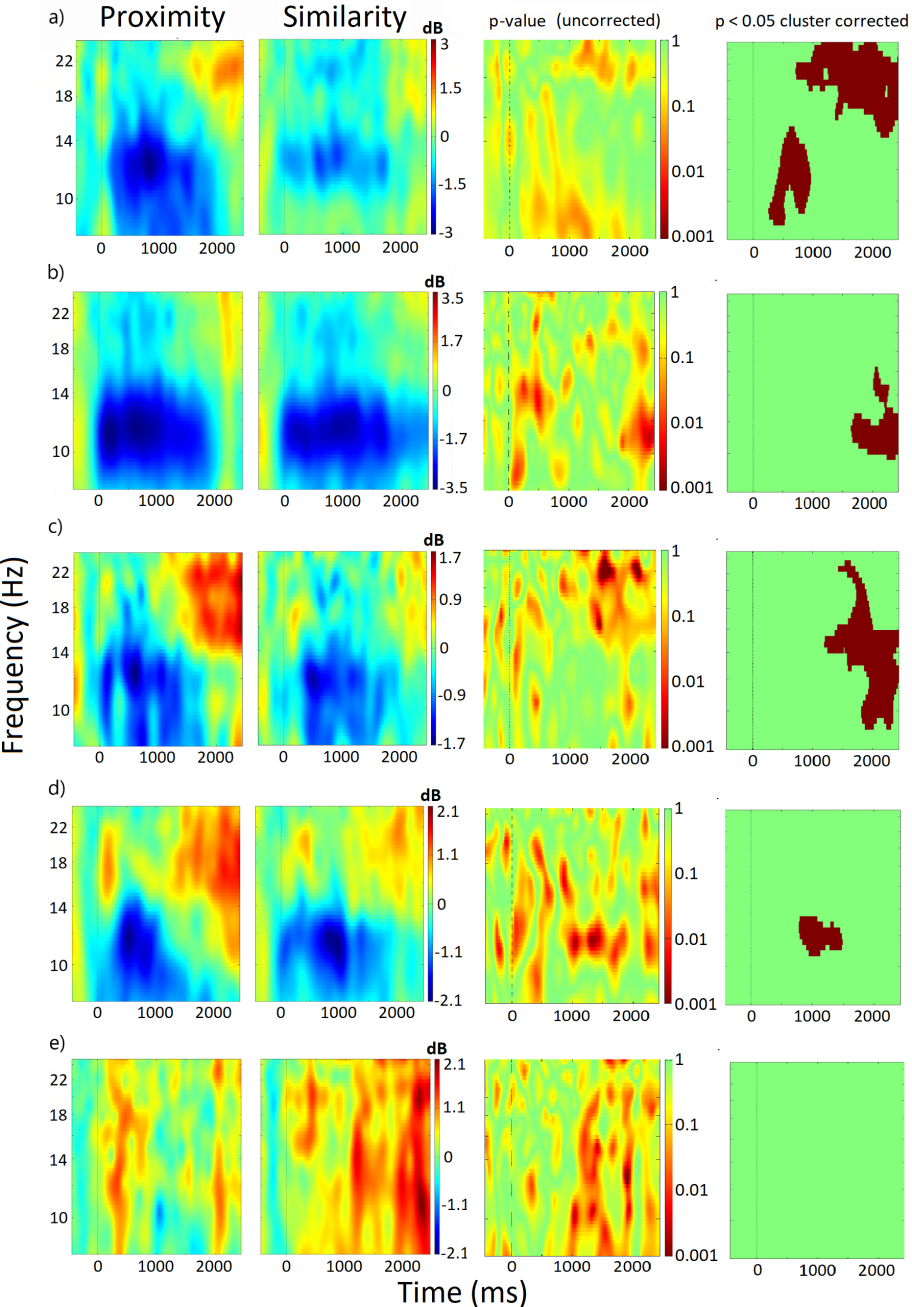
ERSPs of the 5 IC-clusters of interest. Mean log-ERSPs (8–25 Hz) relative to stimulus onset (0) of the IC-clusters of interest for proximity (first column) and similarity (second column) conditions in the orientation detection task, along with uncorrected (third column) and cluster corrected (fourth column) *p* values. From top to bottom: a) left sensorimotor, b) right sensorimotor, c) left parietal, d) right parietal, e) occipital.

To quantitatively analyze differences in oscillatory activity between proximity and similarity grouping epochs, ERSPs were plotted under both experimental conditions (see 3.3 Section) along with statistical differences between them. Four (left sensorimotor, right sensorimotor, left parietal and right parietal) out of five IC-clusters analyzed showed statistically significant differences between the two grouping conditions in ERSP during the time epoch analyzed. The occipital IC-cluster did not show significant differences between conditions in ERSP. See Fig 4.

Fig 4A shows the ERSPs of a cluster of 16 ICs located in or near left sensorimotor scalp sites. Mean ERSP activity of the left sensorimotor IC-cluster for epochs in both proximity and similarity conditions shows an alpha band event-related desynchronization (ERD) time-locked to the beginning of the haptic exploration that was significantly more pronounced in the proximity condition within the 500–1000 ms time window. ERSP activity for this cluster also reveals an ongoing ERD in the beta band peaking around 20–22 Hz that was significantly more pronounced under the similarity condition. Last, proximity condition showed a greater event related synchronization (ERS) relative to similarity condition that starts approximately after the response execution (1500 ms).

Fig 4B shows the mean scalp map, ERSP and power spectra of a cluster of 18 ICs, located over the right sensorimotor cortex. Visual inspection of ERSP activity revealed an ERD in alpha and beta bands in both proximity and similarity conditions time-locked to the onset of the exploration. In addition, the statistical analysis showed that the alpha band ERD lasted significantly longer in the similarity than in the proximity condition.

Results from left parietal IC cluster (17 ICs) are displayed in Fig 4C. Both conditions exhibit an alpha band ERD that also extends to beta band. No differences in ERD between grouping conditions appeared, but the proximity condition showed an earlier and more pronounced resynchronization after ERD in both alpha and low beta bands.

ERSP from the IC cluster located over the right parietal cortex (20 ICs) indicate a greater alpha band ERD in the similarity condition around 1000 ms after the start of the haptic exploration (Fig 4D). Neither differences in ERS intensity nor timing in alpha and beta bands arise in this IC-cluster.

Finally, Fig 4E displays the scalp maps, ERSP and spectral power of an IC cluster located over the occipital cortex (21 ICs). Both conditions showed alpha and beta bands ERS during the task period in both frequency bands analyzed. No significant differences appeared between the experimental conditions.

## Discussion

The present study was motivated by the lack of research considering both behavioral and electrophysiological data on the applicability of the perceptual grouping principles to active touch and their neural correlates. As far as we know, this is the first electrophysiological study to investigate the brain activity underlying perceptual grouping in the haptic modality using spatial proximity and texture similarity grouping principles. The study focused on event-related spectral perturbation, a measure that accounts for the time and frequency domains of the EEG signal [43,51], and, specifically, in two frequency bands: alpha (8–14 Hz) and beta (15–25 Hz), which have been widely related to changes in brain activity in sensorimotor and parietal cortices [52–54]. To avoid confounds arising from the mixture of brain and non-brain activities obtained in scalp-recorded EEG signals, we used independent component analysis to decompose the EEG data into maximally independent components [41], which were further analyzed via time-frequency analysis [43,44]. Overall, there were two main results: (1) Participants detected the orientation of stimuli grouped by proximity faster than those grouped by similarity, and (2) the IC-cluster ERSP analyses revealed a widespread bilateral activation of sensorimotor and parietal cortices, indicating: a) Selective alpha and beta band ERD over both ipsi- and contra-lateral sensorimotor and parietal areas, and a lack of involvement of the occipital cortex in haptic grouping during the orientation task; b) different timing of the alpha ERD/ERS pattern over those areas in the two different grouping conditions, mirroring the behavioral results; c) greater but shorter alpha and beta ERD over contra-lateral sensorimotor cortex in the proximity condition, possibly indicating the existence of different exploration demands centered in global (configurational) and local (individual) properties of proximity and similarity grouping respectively, and d) more pronounced alpha ERD over the right parietal cortex in the similarity condition, that could be related to the more demanding spatial integration processes in this condition. These results and their implications are discussed in detail below.

### Faster responses to stimuli grouped by spatial proximity

As observed in previous visual studies [19], participants responded faster to stimuli grouped by proximity than to those grouped by texture similarity. In the visual modality, this advantage has been linked to the dominance of holistic properties over component properties in the perceptual process [55,56]. According to this view, detecting the orientation of an array of elements (which in fact involves the detection of a global property) that are grouped by spatial proximity is faster because it could be considered as a holistic/configural property that relies on the relationship between individual components. On the other hand, texture could be considered a component property leading to higher RTs. The results of the present study suggest that comparable processes might occur in active touch. In particular, the spatial relation (the different distances) between the elements of the haptic pattern in the spatial proximity condition could also be considered as a holistic or configural feature [55–57]. This feature describes a specific relation among the elements of the array rather than informing about the component itself; in other words, it gives information about the orientation of the pattern without the need to explore each element individually. On the other hand, a component feature like texture requires the identification of each element prior to the integration into a whole percept, thus leading to faster identification of patterns grouped by proximity than those grouped by similarity. While in visual modality the advantage of holistic/configural properties is thought to be related to spatial frequency analysis [58], the differences found in the present study could be related to the different exploration demands in each grouping condition. When stimuli are grouped by proximity, participants can use the different spatial gaps in the global pattern to detect the orientation of the array without the need to identify and integrate the individual (local) characteristics of each element, thus leading to faster responses for stimuli grouped by proximity. By contrast, stimuli grouped by similarity would require the detection of the local component properties (texture) of each individual stimulus and integrate this information into a single object to come up with an answer. However, it is important to note that the proposed similarities between perceptual grouping in vision and haptics should be taken with caution. As we pointed out in the introduction, there are important differences in how the sensory information is acquired by each sensory modality (serial *vs*. parallel). Further investigation, including passive tactile tasks, would be necessary to draw solid conclusions about the commonalities between perceptual grouping in vision and touch.

### Event-related spectral changes associated with different grouping conditions

The ERSP is thought to measure average dynamic changes in amplitude of the broadband frequency spectrum as a function of time relative to the onset of the task [44]. No statistical differences arose between epochs belonging to different conditions during the baseline, so we will only discuss ERSP and spectral power changes during the task period.

#### Left sensorimotor IC cluster

The results of ERSP over the left motor cluster show an ERD in the alpha band that was more pronounced in the proximity condition between 500–1000 ms after the start of the task (see Fig 4A). This ERD was followed by an ERS that started approximately after the end of the task in each condition (1400 and 1800 ms respectively). Decreases in power/amplitude reflecting ERD in alpha band have been associated with high excitability states of the implicated areas [59]. In accordance with this view, the alpha ERD found over the left sensorimotor cluster is thought to be closely linked to active cognitive processing and may serve as an indirect measure of activity in those areas. The involvement of contralateral motor and sensory cortices in a haptic orientation task is not surprising given that these cortical areas, located at contralateral post-central gyrus and pre-central gyrus, have been widely related to somatosensory perception, movement organization, voluntary hand movement, finger proprioception and contralateral finger and hand movements [60]. Thus, the greater (but shorter) alpha ERD in the proximity condition could reflect: (1) the distinct exploration demands required in the two different grouping conditions (see Section 5.1), and (2) the differential exploration and response times of proximity and similarity conditions that would be reflected here in the timing of the alpha ERD/ERS that mirrors the behavioral RTs.

A similar pattern of ERD/ERS emerged from beta band ERSP. While its functional role is not as well understood as other frequency bands, beta-band activity over sensorimotor areas has been related to motor control [61] and the maintenance of the current sensorimotor or cognitive state [62]. Beta band is usually desynchronized with movement and recovers during immobility much faster than alpha rhythms, showing a stronger synchronization after the cessation of movements [63]. In accordance with this view, the differences between proximity and similarity conditions in beta-band activity over the left sensorimotor cortex seem to have a motor origin, possibly related to the shorter exploration times found in the proximity condition, with a transient ERD and a faster resynchronization in this grouping condition that seem to reflect the earlier cessation of the hand movements in this condition. These results are in line with previous findings that linked attenuated beta activity to voluntary movements across the motor-related brain regions, especially in the peri-rolandic region [63,64].

#### Right sensorimotor IC cluster

As seen in the left sensorimotor cluster, the right sensorimotor IC cluster exhibited alpha band ERD lasting significantly longer in the similarity condition, indicating the involvement of the ipsilateral sensorimotor cortex in the grouping task (Fig 4B). ERSP activity within the beta band follows the same ERD/ERS sequence as seen in alpha band, but no statistical differences appeared between conditions in this frequency band. Activity of ipsilateral motor and sensory areas usually accompanies the execution of unimanual tasks [65–67]. However, the purpose of this involvement remains unclear [68]. It could be just a ‘cross-talk’ through projections between bilateral areas that facilitate the movement, or even the reflection of inhibitory processes that prevent involuntary movements [67,69,70]. Moreover, the strength of the involvement of ipsilateral sensorimotor areas correlates with task complexity [71,72]. Thus, the extended alpha ERD found in the similarity condition in this study could be an index of the greater difficulty/complexity of the similarity grouping condition that requires a more intense involvement of the ipsilateral cortex. However, it could also be that the differences arose from the longer exploration times in the similarity condition and the need to engage facilitatory and/or inhibitory processes for a longer period. Interestingly, the scalp topography shows a slightly more anterior location of the IC cluster, possibly involving the pre-motor cortex and supplementary motor area (SMA). Thus, the involvement of the ipsilateral cortex found in this cluster could be related to the online sensory control of the motor sequence and guidance of action. This would explain the ipsilateral beta-band desynchronization, as this activity is usually linked to motor functions [62], information transmission between the cortex and periphery [62,73] and reciprocal connections between muscles and cortex [74]. Overall, the results point to a motor origin of the ipsilateral sensorimotor cluster activity, related to the online control of actions via sensory feedback (including inhibition of ipsilateral and facilitation of contralateral hand movements). This recruitment of ipsilateral motor areas was even greater (or at least longer lasting) in the similarity condition. A possible explanation is the greater requirements in terms of exploratory strategy and integration of individual elements in similarity condition. Alternatively, it is also possible that the greater alpha band ERD found in the similarity condition was a byproduct of the longer exploration times in this condition, an explanation that agrees with the slower RTs found in this condition.

#### Left parietal IC cluster

Modulations of the alpha band SMR during tactile tasks are usually widespread, showing a bilateral pattern that includes parietal regions [28,75] that play an important role in the integration of somatosensory signals [76]. Furthermore, the information flow from sensorimotor to parietal cortices within the alpha frequency range is thought to reflect the transmission of a copy of the efferent motor information essential to sensorimotor integration [77]. The alpha band ERD found in the present study in contralateral parietal areas (Fig 4C) could reflect the cortical integration of the peripheral information acquired by the sensorimotor areas while performing the task. In the similarity grouping condition, with higher integration demands, participants need to explore each individual element, differentiate between two different textures (microspatial component) and integrate this information in order to detect the orientation of the patterns (macrospatial component), leading to greater ERD and later resynchronization [78]. In this line, Roland et al. [79] and Kitada et al. [80] using fMRI, found activation of the parietal cortex during roughness (microspatial) and shape/length (macrospatial) discrimination. In particular, Kitada et al. [80] found increased activation of the parietal operculum and insula (including secondary somatosensory cortex) during a tactile roughness estimation task, two areas that have previously been linked to roughness discrimination [81] and that have direct connections with the primary somatosensory cortex [82]. Finally, Karhu and Tesche [83], providing electrical stimulation to the median nerve, discovered synchronized activity between neurons in primary and secondary contralateral somatosensory cortices (SI/SII). This finding suggests the involvement of SII and other cortices near the parietal operculum in the early processing of the somatosensory input. This finding is in line with the similar activation and timing found in the present study between somatosensory and parietal cortices. However, as we noted in the left and right sensorimotor clusters, we cannot discard the possibility that the increased activity found in the similarity condition was due to the longer exploration times in this condition. However, given that the differences between conditions seem to be due to the greater resynchronization in the proximity condition, an explanation based on the greater integration demands seems more plausible, as smaller resynchronization usually follows greater and more intense cortical activation [59].

#### Right parietal IC cluster

ERSP within the right parietal IC cluster followed an alpha ERD/ERS pattern similar to the one found in the right sensorimotor cortex. This result indicates the conjoint activation of ipsilateral right parietal and sensorimotor cortices as occurs with the homotopic left areas. There has been converging evidence of right hemisphere dominance in spatial processing [84]. Particularly, right parietal activity has been linked to the integration of spatial information and coherent brain activity between parietal and motor cortices, and is thought to reflect the integration of sensorimotor behavior, especially when movements are guided by external haptic feedback [85]. Thus, the increased activity in the right parietal IC cluster found in the present study might be related to the processing of the spatial characteristics of the task and the use of the resultant haptic feedback to guide the exploration movements [86]. The greater activation found in the similarity condition might be the result of the more demanding spatial integration processes required in this condition. The sensorimotor areas of the left hemisphere would use this information to modify the ongoing motor commands [87]. Another possible explanation is related to the role of the parietal cortex in tactile working memory. Li-Hegner et al. [88] found that ipsilateral temporoparietal activity, predominantly around SII areas, contributes to the maintenance of tactile pattern information in working memory. Given the previous findings of SII neurons involved in the perception of both roughness [89] and orientation [90], it is plausible that the differences in alpha ERD in our study were associated with the greater sensorimotor demands of the similarity condition, as participants needed to process both micro-geometrical (integration of information about two different textures) and macro-geometrical (orientation) features [91,92]. In fact, right-hand discrimination of micro-geometrical features specifically activates the right angular gyrus [78], which is in line with the greater activation of the right parietal IC cluster in the similarity condition.

Finally, we cannot rule out the possibility of an attentional origin of at least part of the right parietal activity. The right temporoparietal junction (TPJ), as part of the ventral frontoparietal attention network, is actively involved in reorienting attention to salient stimuli [93]. Thus, it could be argued that patterns grouped by similarity entail a stronger attentional capture and/or focus, due to the presence of two different textures and the higher processing demands. However, while it is difficult to disentangle attention and working memory processes due to their functional overlapping [94], it is unlikely that the activity found in our study had an attentional capture origin, given that the alpha ERD in the similarity condition peaked around 900–1000 ms after the first contact with the tactile pattern, whereas activity derived from attentional capture would be confined to the first few hundred milliseconds after the onset of the task.

#### Occipital IC cluster

Analysis of the ERSP of the occipital IC cluster revealed not only the absence of alpha and beta band ERD but also the existence of ERS at occipital areas in both grouping conditions. This is consistent with a brain state of reduced information processing (‘idling’ state) and active inhibition that occurs within brain areas that are not relevant for the task [95,96]. Given that our participants performed the haptic task with their eyes open, the significant synchronization over occipital areas may reflect inhibition of irrelevant visual processing throughout the task. This appears to be inconsistent with studies that found activation of visual areas during haptic perception [29,97]. However, the implication of visual areas in haptic perception is related to macroscopic features such as object shape or 3D structure [30,97] as well as to object recognition [98]. In the present study, the detection of orientation relied on micro-geometric features like texture discrimination and the spatial relation between scattered elements that did not conform to a familiar or a shape-defined object, a process that would not engage the visual cortex [99].

Taken together, our results show the involvement of a bilateral network of sensorimotor and parietal areas in detecting the orientation of Gestalt grouped patterns in haptic modality. This is in accordance with previous haptic modality studies addressing orientation [78,100] and texture processing [79,101]. These results are also in line with hemispheric specialization views that linked left hemisphere activity within sensorimotor and parietal areas (at least in right-handed participants) to movement organization and selection, and the integration of sensorimotor information; and right hemisphere activity to the use of sensory feedback to guide movements and to the process of the spatial components of sensorimotor processing, including those related to attentional orientation and working memory (for a review of hemispheric specialization and integration see [86]). This view is in line with our electrophysiological results, linking the differences in alpha and beta band ERD/ERS in the left (contralateral) cortex to the control of voluntary movements and the integration of sensory information, and the differences in the right (ipsilateral) cortex to the sensory-based guidance of the movement sequence and the spatial aspects of the task, including the integration of spatial information, maintenance of the information in working memory and orientation of attention.

#### Shortcomings, limitations and future directions

To the best of our knowledge, this is the first study to investigate the neurophysiological correlates of two key grouping principles in haptic perception. In this study, we used a more whole-hand exploration task instead of just a one-fingertip exploration task. The haptic task was performed using a novel haptic device that resembles those used for visual perception. However, the study has some limitations that must be addressed in future research. First, the total number of trials was limited. This was due to the needed to manually configure the haptic pattern between trials by placing the cylinders one by one into the appropriate sockets before the beginning of each trial. Further improvements in the novel *MonHap* device would enable us to automate this process, allowing a considerable increase in the number of trials. This would yield reliable EEG recordings with more complex designs. Second, the serial nature of the haptic modality and the extent of the exploration times made it impossible to record evoked activity in addition to spectral ERSP activity, a limitation that is common in tactile experiments. Third, future studies should investigate the functional and causal connectivity between brain areas implicated in haptic perceptual grouping. We expect to observe an intense connectivity between active sensorimotor and parietal areas within and between hemispheres as shown in other haptic studies [28,43]. Finally, our study did not address the question of how the grouping principles interact when two or more congruent or incongruent principles are present within the same stimulus. It would be interesting in future studies to include two or more grouping principles within the same trial. This would allow us to explore the pattern of facilitation and interference, as well as the dominance of one principle over another. This kind of interaction study, together with variations in the relative strength of each grouping principle, could provide a deeper understanding of the process of perceptual grouping in touch and the differences and commonalties with other sensory modalities.

## Conclusions

To conclude, the present study replicates in the haptic modality behavioral findings in the visual modality, showing faster RTs for the stimuli grouped by proximity than for those grouped by similarity. Moreover, the analysis of ERSP activity shows the involvement of a bilateral network of parietal and sensorimotor areas in the processing of the grouped patterns, as indicated by the desynchronization of alpha and beta frequency bands during the task. From the analysis of the differences between epochs in which haptic patterns were grouped by proximity and by similarity, we can conclude that similarity grouping is related to more intense spatial integration processing due to the need to integrate micro-geometrical properties (texture) of the stimulus in order to extract the macro-geometrical properties. This leads to greater processing requirements and more activity in areas implicated in the integration of sensorimotor information and spatial processing. Finally, the absence of reliable activation in occipital areas signals the lack of involvement of visual cortex in haptic perceptual grouping, at least when the task involves only the processing of low level features such as roughness and orientation.
